# Visualization and biopsy of an appendiceal sessile serrated lesion during endoscopic retrograde appendicitis therapy

**DOI:** 10.1055/a-2589-1229

**Published:** 2025-05-14

**Authors:** Guodong Dai, Lujie Chang, Na Chen, Xingfang Jia, Xianyong Cheng

**Affiliations:** 1 562131 Department of Gastroenterology, Binzhou Medical University Hospital, Binzhou, China; 2 562131 Department of Pathology, Binzhou Medical University Hospital, Binzhou, China

A 67-year-old man presented with pain and tenderness in the right lower quadrant. Abdominal computed tomography (CT) scan was performed, which revealed appendicitis and appendiceal fecaliths ( Fig. 1 ). The patient was admitted to our hospital for endoscopic retrograde appendicitis therapy (ERAT). The procedure was performed using a 3.2-mm biopsy channel colonoscope (CF-H290I; Olympus) with successful intubation of a single-use subscope (9-Fr eyeMax; Micro-Tech) into the appendiceal cavity. The subscope demonstrated that the cavity wall of the appendix lumen was slightly hyperemia and edema, and appendiceal fecaliths were found within the appendiceal lumen ( Fig. 2 **a** ). Plenty of normal salines was used to rinse out the fecaliths ( Fig. 2 **b** ). Subsequently, a 1.2-cm laterally spreading tumor was identified within the appendiceal lumen, characterized by a rough, granular mucosal surface covered by a mucous cap ( Fig. 3 **a** , Video 1 ). Methylene blue staining revealed a clear boundary ( Fig. 3 **b** ). A biopsy was performed. The pathological findings of the biopsy were sessile serrated lesions (SSLs) ( Fig. 4 ).

**Fig. 1:**
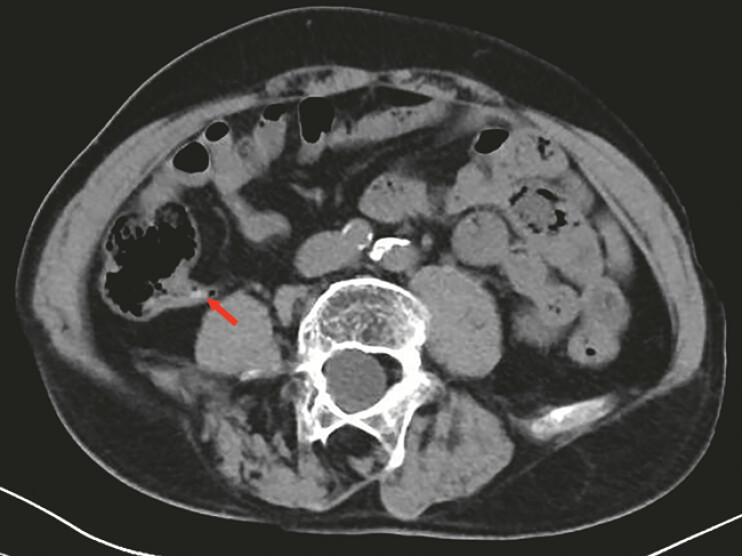
The abdominal CT revealed appendicitis and appendiceal fecalith (the red arrow).

**Fig. 2:**
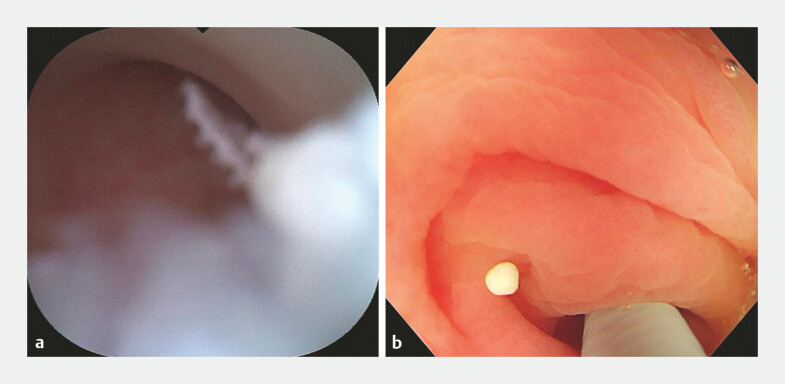
**a** The appendiceal fecaliths in the appendix lumen. **b** The appendiceal fecaliths were rinsed out.

**Fig. 3:**
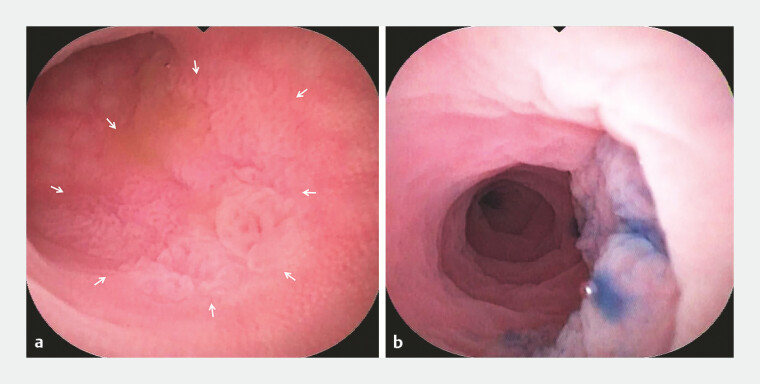
**a** The lesion was an area of rough, granular mucosa (the white arrow) in the appendix lumen. **b** Methylene blue staining showed a clear boundary.

The detection and biopsy of the lesion confined to the lumen of the appendix by the single-use subscope imaging system during endoscopic retrograde appendicitis therapy.Video 1

**Fig. 4:**
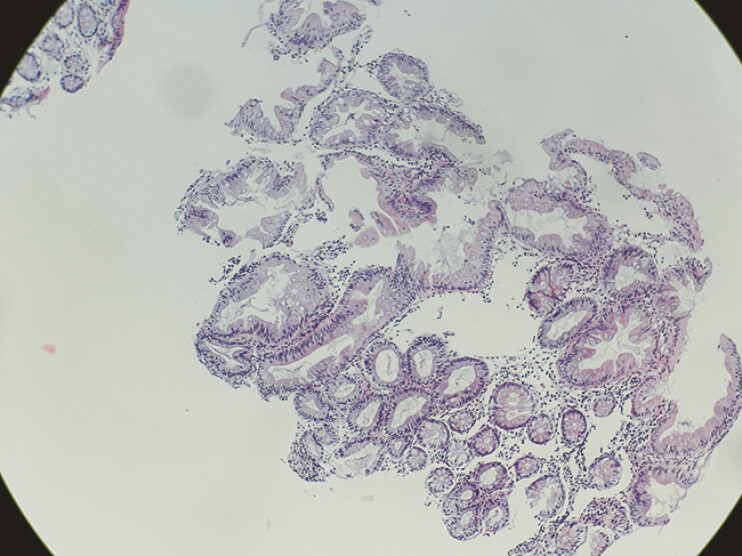
Biopsy pathology of the lesion.

After obtaining informed consent, a laparoscopic appendectomy was performed. During the procedure, it was observed that the appendix was elongated and exhibited mild adhesions to the surrounding tissues. The rough area in the appendiceal lumen was obvious at postoperative specimen ( Fig. 5 **a** . The presence of SSL with low-grade dysplasia was confirmed by the postoperative histopathological analysis ( Fig. 5 **b** ).

**Fig. 5:**
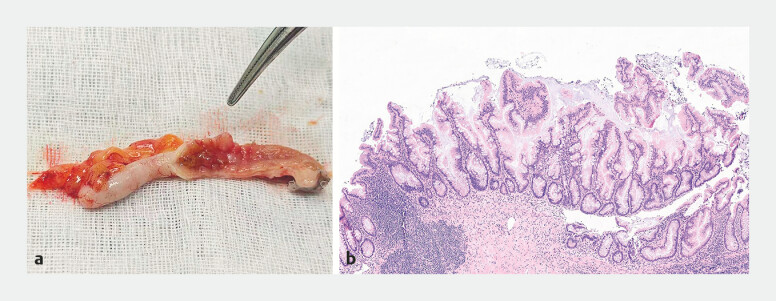
**a** The rough area (the vascular clamp pointed) in the appendix lumen at the removed appendix. **b** Pathology of the lesion in the appendix lumen.

Postoperatively, the patient was given antibiotics therapy and fasted for 3 days. He recovered quickly from mild abdominal pain and was discharged 4 days later. The symptoms of right lower quadrant pain and tenderness were significantly relieved.

Appendiceal SSLs are lesions with malignant potential, they are rarely reported and mainly incidental findings in appendectomy specimens 1 2 . This case demonstrates the utility of the single-use subscope imaging systems in managing appendicitis and detecting appendiceal lesions, providing clear visualization and enabling precise diagnosis and treatment. It provides a possible technique for the early detection and pathological diagnosis of lesions confined within the lumen of the appendix.

Endoscopy_UCTN_Code_CCL_1AD_2AB

## Notes

Endoscopy E-Videos https://eref.thieme.de/e-videos
*E-Videos* is an open access online section of the journal *Endoscopy* , reporting on interesting cases and new techniques in gastroenterological endoscopy. All papers include a high-quality video and are published with a Creative Commons CC-BY license. Endoscopy E-Videos qualify for HINARI discounts and waivers and eligibility is automatically checked during the submission process. We grant 100% waivers to articles whose corresponding authors are based in Group A countries and 50% waivers to those who are based in Group B countries as classified by Research4Life (see: https://www.research4life.org/access/eligibility/ ). This section has its own submission website at https://mc.manuscriptcentral.com/e-videos .
